# Images of women breastfeeding in public: solitude and sociality in recent photographic portraiture

**DOI:** 10.1186/s13006-018-0194-5

**Published:** 2018-12-06

**Authors:** Fiona Giles

**Affiliations:** University of Sydney, Sydney, Australia

**Keywords:** Breastfeeding in public, Images of breastfeeding, Breastfeeding photography, Photographic portraiture, Breastfeeding selfies, Brelfies, Intimate publics

## Abstract

Contemporary images of women breastfeeding — from breastfeeding selfies to fine art — celebrate breastfeeding outside the home by displaying visual records of these occasions to a wider audience. From brelfies posted by celebrities and ordinary parents on social media, to the photography of Tara Ruby and Ivette Ivens, media coverage of lactivist nurse-ins, or fine-art works by Ashlee Jenkins and Sky Boucher, the repertoire of breastfeeding images in developed Western nations has grown and diversified exponentially in the past ten years. A subject that was once the province of religious painting, ethnography, public health advocacy or obscure corners of pornography, is increasingly made visible within the everyday, not only through self-portraiture on social media but also through the work of celebrated photographers and visual artists.

Despite this, there is still an absence of images of women breastfeeding in social circumstances, suggesting a reluctance to make the leap from understanding breastfeeding as a solitary activity, regardless of the space the mother inhabits at the time, to a companionable behaviour integral to our social landscape. Images predominate of women breastfeeding alone, or at best with other breastfeeding women, revealing a further binary dividing the acceptable from the unacceptable, where the private vs. public has been conflated with the solitary vs. social.

This article provides a textual analysis of contemporary photographic portraiture to interpret the meanings of key works, and their patterns of signification. It asks to what extent these images advance efforts to normalize breastfeeding and make it publicly commonplace, or reinforce unhelpful binaries, using an iconography based on the religious origins of portraiture itself: the virtuous, devoted mother, unaccompanied but for her child. I conclude that the lack of images where breastfeeding women are integrated into social occasions is partly due to the lack of opportunities for women to breastfeed socially, and few motives for these instances to be recorded, and that there is an unspoken proxemics of viewing space yet to be traversed.

‘The social does not “influence” the private; it dwells within it.’ (Russell Jacoby, 1973)

‘The personal is the general. Publics presume intimacy.’ (Laurent Berlant, 2008)

## Background

The challenge to bring breastfeeding into public consciousness, space and place in Western culture has been tackled for the past several decades through a variety of public policy initiatives and media campaigns. These include legislation protecting women’s right to feed their children when they are hungry, regardless of location; fines for preventing women from breastfeeding in public, the creation of ‘nursing nooks’ or ‘Portable Lactation Modules’ where women are encouraged to take their children to breastfeed when outside the home; ‘Breastfeeding Welcome Here’ designated cafes and Baby Café services; and life-size cardboard cut-outs of images of breastfeeding women placed in public buildings, businesses and restaurants in the UK, US and Canada [1–6]. Added to these efforts are the nurse-ins held at public venues following occasions when women have been ejected for breastfeeding, the annual *Guinness Book of Records* event for women nursing at one time together, and social media activism such as #normalizebreastfeeding, or #thisishowwebreastfeed, as well as Facebook pages and Instagram accounts devoted to breastfeeding support.

While all of these contribute to the different layers of breastfeeding positive discourse, efforts to encourage women to feel safe breastfeeding in public — which for the purpose of this essay, I take to mean outside the home — have had mixed success. UK mothers in particular, but in many countries, still struggle to find support from the general population [7, 8]. The majority of studies across a range of countries and demographics provide similar findings: breastfeeding in public is ‘acceptable’ to a majority of adult men and women if performed ‘discreetly’, either covered or obscured by clothing or furniture, or out of sight in secluded or designated parenting or nursing spaces [6–10]. A majority of adult men and women still regard women openly breastfeeding where their children need them to, without cover, as a sign of inadequate parenting or maternal exhibitionism [11–13]. With fewer than 50% of women in developed nations exclusively breastfeeding to 6 months, as recommended by WHO, and as few as 1% of mothers achieving this in the UK, the ability of mothers to feed their children in diverse locations may provide significant scope for improvement [14].

For good reason, then, mothers continue to feel anxious about their ability to breastfeed safely outside domestic settings. However, it needs to be acknowledged that breastfeeding in domestic settings may be social, and public breastfeeding is often solitary, so that the critical element that creates tension and vulnerability, is when an audience is invoked, regardless of the mother’s location. While breastfeeding in spaces beyond the domestic invites a higher level of scrutiny and standard of decorum, the element of solitude that inevitably accompanies those occasions adds to her sense of conspicuousness and pressure to conform to those standards, whether real or imagined. In a real sense, then, regardless of place, it is the presence of a possible or real audience that adds to the challenge for many breastfeeding mothers.

Mothers who recognize they’re entitled to feed publicly, thus feel that they need competence borne of experience to nurse ‘discreetly’ before they are willing to try [2, 15–18]. Building confidence while nursing under scrutiny adds to their disincentives [2, 7]. Additionally, it is arguable that media coverage of incidents when a woman has been ejected from a public venue for breastfeeding may add to the anxiety new mothers feel about breastfeeding in public. While 72% of women polled in a UK study state their acceptance of breastfeeding in public, 60% are fearful of harassment, disapproving looks, or being asked to desist or leave [19]. While media coverage of nurse-ins and lactivism (breastfeeding activism on social media) usefully raises awareness of women’s right to breastfeed in public, and keeps the issue of women’s right to breastfeed on the public agenda, it may also reinforce the belief that breastfeeding is a contested behaviour that invites scrutiny, if not disapproval, and contributes to the disconnect between actual and perceived disapproval of public breastfeeding [18–22].

In addition to caring for their children while outside the home, breastfeeding mothers also feel responsible for the feelings of others, and out of place, as Boyer has shown: both alienated and under surveillance [7]. In such cases, responsibility for their offspring competes with a sense of responsibility for social harmony, and for the management of their lactating body as an acceptable presence in human company. Through this process of alienation, the moral authority of the mother in relation to her child’s needs is undermined by the competing moral authority of any public spaces she inhabits. In addition to undermining breastfeeding rights in general, this represents an important psychosocial injury to the maternal subject, who, as Ruddick argues in *Maternal Thinking*, is responsible for the social training of her offspring. To undermine the authority of the mother is to undermine an important aspect of her maternal role as instructor and mentor, additional to her role as provider and nurturer [23]. As the woman practicing motherwork is banished from view or shamed into invisibility, her maternal status in general is diminished. To use Berlant’s phrase, it could be said that to see breastfeeding in public, whether via an image or the presence of a mother nursing her child nearby, is to see enacted ‘the historical antimony between women and public authority’ [24].

## Breastfeeding images, intimate publics and the role of sociality

Understanding the role of breastfeeding images in the construction of what Berlant calls ‘intimate publics’ is useful to an understanding of how these images might assist in breaking down this antinomy. How might a broader variety of images of women breastfeeding encourage mothers to breastfeed openly, for longer, and both inside and outside the home? And if images can so encourage women to breastfeed, do different kinds of images bear different potentials in achieving this end?

Intimate publics are defined by Berlant as affective spaces within which ‘a sense of belonging to a community’, or a shared ‘emotional knowledge’, is represented [25]. The concept of intimate publics has been adopted within social media studies to describe an enabling space within which women or other marginalized communities can communicate their shared experiences. However, it needs to be acknowledged that the term, as developed by Berlant, bears an ambivalent relationship to social change, with its capacity to both dismantle and reinforce subjugation through its collusion with what she calls a politics of sentimentality. Berlant warns, any ‘intimate public’s relation to the political and to politics is extremely uneven and complex... [since] What makes a public sphere intimate is an expectation that the consumers of its particular stuff already share a worldview and emotional knowledge that they have derived from a broadly common historical experience’ [25]. Primarily at work within women’s popular culture, such intimate publics provide a way of ‘experiencing one’s own story as part of something social’ [26]. Nevertheless, this mostly exists ‘only in *proximity* to the political…acting as a critical chorus that sees the expression of emotional response and conceptual recalibration as achievement enough’ [26].

To what extent then, might popular forms of breastfeeding portraiture — such as brelfies and glamour shots — be only proximate? To what extent might they be nearby to, without engaging with, political change? In addition to the uncertain value of images that circulate within intimate publics, the nature of the breastfeeding images in circulation also warrants analysis, since they almost exclusively depict a breastfeeding mother alone with her child. This means that even where breastfeeding images are circulated throughout the many and varied digital publics of social media, or framed for exhibition in galleries or homes, and regardless of the space within which the mother feeds her child when photographed, they consistently reinforce breastfeeding as solitary behaviour. It could be said then, that the images may fall short in two respects: as participating in an intimate public which is proximate to activism; and as images which represent breastfeeding at a proxemic distance from the social.

While some photographs also depict mothers’ groups in cafés and parks, or a mother feeding a baby with one or two siblings present, it is rare to see adult non-breastfeeding company, or older children, in visual representations of breastfeeding. One exception to this may be achieved when a nurse-in is reported in mainstream, local or social media, or photographed by a professional breastfeeding photographer for a breastfeeding community website. Nevertheless, these images are, by definition, also framed as distant from the everyday, since they represent a protest group formed for the occasion of the protest, which is itself a theatre of difference designed to rupture ordinary civic interaction [20]. The proxemic distance between the breastfeeding mothers and their audience is symptomatic of this tension between the desire to celebrate breastfeeding as best maternal practice and the anxiety of rendering it into spectacle. While nurse-in photographs perform spectacle as protest, they also detract from the embodied breastfeeding relationship of the individual mothers, who are subsumed into the group. There is strength and safety in numbers, but there is also anonymity.

For this reason, I propose that a second binary exists alongside the more familiar public/private binary that polices breastfeeding. Not only is ‘private’ breastfeeding considered more acceptable, but so too is solitary breastfeeding.^1^ In both cases the common element is discretion, if not invisibility, together with strict codes of decorum that conceal the woman’s body, in particular her nipples. Being inside or outside the home is not the point; rather it is the bringing into being of an audience that makes breastfeeding controversial. It is the relational economy that this signifies of the mother — with other children, family, friends or strangers — that reminds us of the relational economy at the heart of the breastfeeding act: the mother and her breastfeeding child. By shifting the problematic from one that positions breastfeeding in private as opposed to breastfeeding in public, I suggest we dismantle the private/public binary and begin to think of breastfeeding in terms of a continuum from solitude to sociality, encompassing the variety of relational economies that this offers [27, 28]. It is here that images of women breastfeeding while engaging socially with non-breastfeeding others — whether inside or outside the home — might have the most value; and where proxemic distance may begin to be traversed.

## Contemporary images of breastfeeding: an overview

Several decades of bottle feeding throughout the twentieth century contributed to uncertainty about breastfeeding decorum alongside ignorance of its benefits and normality. Knowledge of breastfeeding skills and behaviours faded. There were few breastfeeding images in circulation to assist, and formula advertising began to dominate the infant feeding imaginary [29]. There simply weren’t enough mothers visibly breastfeeding throughout most of the twentieth century for contemporary breastfeeding images to become commonplace; and standards of feminine modesty forbade exposure of breasts in general. Together with the decline of breastfeeding throughout much of the twentieth century, the rising phenomenon of the stay-at-home mother also meant that the minority who did breastfeed had fewer reasons to leave the house with their young children [30].

It should not be surprising, then, that images of breastfeeding that emerged in breastfeeding promotion campaigns following the 1980s drew primarily from Renaissance paintings of the Madonna and child, or nineteenth century domestic depictions, such as those by Picasso or Cassatt, where breastfeeding mothers are represented alone at home or, if in any company at all, with other young children. It is perhaps not insignificant that the genre of portraiture itself became popular on the basis of Madonna and Child paintings during the Renaissance; and it could be argued that *Maria Lactans* images are embedded in the very idea of representing an individual as worthy of being painted [31]. Thus the images of women breastfeeding their children that emerged from post 1980s advocacy campaigns would have been considered suitably genteel in a period when breastfeeding was only just becoming socially acceptable.

Yet late Victorian and early twentieth century photographs indicate that breastfeeding in public was in previous times unremarkable, with photographs showing women breastfeeding in mixed groups outside the home during the Great Depression, working women in social circumstances nursing toddlers — including a woman tandem nursing a baby and bear cub — and wet nurses in hospitals donating their milk [31–33]. Images of garments designed for mothers to breastfeed in public have also been preserved from the Victorian era [34]. However, these have only been displayed through websites in recent years, so that the portrayal and promotion of breastfeeding as a preferred maternal behaviour has mostly tended towards the conservative and familiar.^2^

Due to the virtual invisibility of breastfeeding throughout the second half of the twentieth century, then, advocacy images from the late twentieth century followed existing populist iconography, producing images of a mother alone with her baby in a domestic setting, her gaze fixed on the infant and her breasts and milk hidden from view by the child’s body, and the mother’s clothing and arms. Widely circulated celebrity images at the turn of this century, such as Jerry Hall breastfeeding on the cover of *Vanity Fair* in1998, and Lucy Lawless posing for a New Zealand World Breastfeeding Week poster in 2001, conformed to this tradition, despite the feminist credentials of these two mothers and their high-profile media identities. While Jerry Hall returns the gaze of the viewer and is more overtly positioned as powerful, they both remain securely embedded within the *Maria Lactans* tradition which sanctifies the breastfeeding mother as dutiful and asexual, breastfeeding alone, within a domestic setting.

As advocacy discourse and breastfeeding has increased, the media in general has continued to shy away from images of breastfeeding mothers, with a 2004 study finding that only 1.3% of news stories were accompanied by an image of a breastfeeding mother [35–37]. Both squeamishness about breastfeeding and uncertainty about breastfeeding protocols contribute to this lack of image-making on the part of news media, together with a persistent gender bias against representing women in the news generally [38]. Caution around images of breastfeeding was evident in 2015 when the Australian edition of *Elle* magazine featured the model Nicole Trunfio breastfeeding her four-month-old son Zion — but was released only to subscribers and withheld from newsstands, which instead featured a similar image, showing Zion sleeping. The editor explained that the subscriber issue was meant as a special ‘thank you’ to its regular readership but also generated disappointment among some readers saying the image deserved wider release in the interests of normalizing breastfeeding [39]. Perhaps the furore following the *Time* magazine cover image in 2012 showing a three-year-old boy breastfeeding while standing on a chair, both mother and child returning the viewers’ gaze, contributed to the editor’s nervousness.

The publication of successive breastfeeding portraits has been controversial, for example, Skye Boucher’s ‘Tea for Two’ showing her breastfeeding her twins on Tumblr [40]. There is still commercial and critical risk associated with breastfeeding images within both mainstream media and online spaces with niche audiences. And dominant images reinforce an association between breastfeeding and maternal virtue that is domestic and solitary, with the mother depicted alone at home. While this is in itself not an unrealistic image of breastfeeding, it is by no means the only one, yet it remains dominant despite the rise of the brelfie (or breastfeeding selfie) which has enabled breastfeeding self-portraiture, framing the mother’s face, breast and baby, from her own perspective, in a wider variety of locations.

Unsurprisingly, brelfies too, have tended to represent women alone, since a selfie, by definition, is a form of self-portraiture that highlights the selfie-taker, and its popularity derives from the autonomy of the photographer who no longer needs a companion to have her photo taken.^3^ Although social media has enabled the development of digital publics within which to share these images, they too are predominantly images of women alone with their children in domestic settings. Nevertheless, brelfies provide an important consciousness-raising function, and have added a diversity of images to assist women in understanding that their own breastfeeding behaviours and bodily appearances are widely shared [40]. Brelfies also provide women with an awareness that images of themselves breastfeeding can be gestural invitations to others to see them breastfeeding, as well as documenting their own experiences for posterity, much as they would their wedding photos, or their child’s first smile [41, 42]. This gestural invitation to be seen is in and of itself an important advance on previous ideals of seclusion and invisibility. Brelfies have therefore made an important contribution to socializing breastfeeding through the digital sharing of their images [27, 28].

In considering the limitations of brelfies in promoting public, or socialized, breastfeeding, Berlant’s concept of intimate publics is also useful, since while brelfies provide an accessible communicative tool for women to build confidence within their social groups, the images have themselves tended to reify the domestic and solitary breastfeeding mother, alone with her child. Although some breflies are taken in outdoor settings and cafes, the women are still in most instances alone, and solitude is the basis for most selfies. This tends to reinforce, rather than depart from, the debates about public breastfeeding propriety. When brelfies have been reported on by women’s mainstream media, the stories have been situated within the context of debates around visibility and breastfeeding, expressing the same doubts that studies regarding breastfeeding in public have identified: is there really a need to breastfeed outside the home; and shouldn’t mothers do it discretely if they must do it at all? In reporting on controversies in response to posting brelfies, the questions run in parallel: are the women being narcissistic by recording their breastfeeding moments; and is it ‘naked exhibitionism’ to post them online? [43].

In summary, although brelfies contribute to the communicative potential of breastfeeding images, they are still confined to a virtual community which simultaneously expresses and limits its potential. As Berlant explains, while an intimate public creates a ‘sense of belonging’, it exists in a ‘politico-sentimental’ sphere which ‘seeks out the monumental time of emotional recognition, a sphere of dreaming and memory, and translates that sense into an imaginary realm of possible acting’ which is deeply ambivalent to change [44]. Seductively self-reinforcing and supportive, it may still not invite breastfeeding mothers to participate in open, mixed society while breastfeeding. And it may reinforce rather than dismantle the stagnant and resistant terms of the debate.

## Commercial breastfeeding portraiture

Breastfeeding portraiture has developed alongside the brelfie phenomenon, but links to an older tradition of fine art photographic portraiture, reaching as far back as photography itself. In its contemporary iteration, breastfeeding portraiture is perhaps a natural extension of the pregnancy portraiture industry, beginning with the celebrity photograph by Annie Liebowitz of Demi Moore on the cover of *Vanity Fair* in 1991and followed by Cindy Crawford on the cover of *W Magazine* in 1999. Other cover images have appeared since then, including Brooke Shields for *Vogue* in 2003, Britney Spears for *Harper’s Bazaar* in 2006, Christine Aguilera for *Marie Claire* in 2008, Mariah Carey on *Life and Style* in 2011, Jessica Simpson on *Elle* in 2012, Megan Gale on *Marie Claire* Australia in 2014 – and coming full circle — Selena Williams for *Vanity Fair* in 2017. Numerous other examples have appeared in social media, such as a pregnant Beyonce amid flowers and in fashion shoots (2017) and Alanis Morisette under water (2016).

The idea that the naked pregnant body could be linked to fashion and beauty may have negative consequences for women who feel challenged to live up to the high standards set by actresses, musicians and supermodels. On the other hand, the appearance and celebration of the naked pregnant body as an aesthetic object also offers women a more positive public image within which to frame their pregnancy, boosting their identity as mothers and the many shapes women’s bodies adopt throughout their lifetime. It was not long before the same celebrities began breastfeeding their children, and being in the image-making business, photos of breastfeeding celebrities were soon widely circulated, such as Angelina Jolie breastfeeding on the cover of *W Magazine* in 2008, taken by her then-husband, the actor Brad Pitt, and Miranda Kerr breastfeeding on her skincare website in 2011. In the same year, Daniel Edwards’ bronze sculpture of Jolie, *Landmark for Breastfeeding*, was unveiled, depicting her in life size, naked and seated, tandem nursing her twins.

The proliferation of celebrities breastfeeding not only attracted mainstream media to the brelfie phenomenon, but also led the way for women to seek out professional breastfeeding portrait photographers. It is curious to see how this pro-social celebrity trend might have encouraged women to record these moments as worthy of belonging to their personal archives, and to sharing their images with friends on social media, particularly following the more lenient policy toward breastfeeding images adopted by Facebook in 2015 after lactivist protests [27].

In the realm of commercial breastfeeding portraiture, a double function emerges, with extensive websites and social media accounts showcasing glamorous portraits of breastfeeding mothers and children, at the same time that the subjects may purchase the photo for their own use. From the perspective of bringing breastfeeding images into digital media circulation, these sites also participate in the construction of an intimate public, displaying exquisite images celebrating breastfeeding to an audience largely consisting of other breastfeeding women. This photographic genre is also distinct from activist breastfeeding photography, installations, and artworks, which adopt a provocative stance in relation to their audiences in order to stimulate dialogue. As Buller writes in her analysis of Ashlee Wells Jackson, Jess Dobkin and Jill Miller, such artists work within a tradition that draws from Patty Chang’s and Catherine Opie’s work — or, to reach back further, Mary Kelly’s *Post-Partum Document* (1973–1979) — which renounces the decorative elements of conventional femininity in favour of stark realism, humour and parody [45].

There currently appear to be two sub-genres in this field of commercial photography, the first exemplified by the work of Tara Ruby and Ivette Ivens, which are wholeheartedly glamorous and, to a large extent, idealizations; and secondly, the Normalize Breastfeeding site run by Yvette Michelle and the Whole Mother Center, and the Honest Body Project produced by Nicole McCain. The latter have strikingly different aesthetics: the Honest Body Project uses minimalist scenery and clothing in order to foreground the women’s and children’s bodies; and Normalize Breastfeeding features everyday images of women in suburban settings. The following section of this article provides a close analysis of photographic work by Ivens, Ruby, Michelle and McCain, considering their relationship to or departure from traditional breastfeeding images that reinforce the public/private binary and breastfeeding as a solitary activity.

## Ivette Ivens

One of the most successful photographers in this field, Ivette Ivens has published a collection of breastfeeding photos in print form, *Breastfeeding Goddesses* (2015), is working on a second collection, and curated the Chicago exhibition ‘I breastfeed my toddler’ the same year. Iven’s work has featured in numerous women’s magazine spreads, including US *Cosmopolitan*. Quoted in an interview with *Huffington Post*, Ivens says, ‘“I believe that the more people look at these photos, the more they’ll understand that breastfeeding is a natural thing, and it’s nothing to be ashamed of… I want people to become comfortable with this”’ [46]. Ivens explains that her photographs emphasize the mythic meanings inherent in breastfeeding, stating in another interview that her photographs illustrate “‘the way each woman feels while nursing: pure, beautiful, saintly, celestial”’ [47]. Her images have a fairy tale quality, which, while slightly otherworldly, draw strong links between the continuities between the natural settings and the women’s relationship with their offspring while feeding.

Ivens’ photos have the production values of fashion shoots, with lushly natural or strikingly dramatic locations, exquisite lighting and theatrical costuming reminiscent of a *Vogue* aesthetic along the lines of Grace Coddington’s more impressionistic fashion spreads. The women are styled to perfection, dressed in garments of a texture and hue belonging to the Romantic era. The images showcase the mothers’ power within a visual drama, embodying an ideal of heroinism. The nudity of mother and baby is also minutely staged, such as the image of an African American woman ankle-deep in the sea, wearing nothing but a loose fitting, sheer dress coat, her naked child perched on her leg, also naked, their skin-to- skin contact emphasized by a decorative band just below the woman’s knee and a flower in the child’s hair. They are framed by a misty cityscape, suggestive of a luxury coastal resort. In another shot, a strawberry blonde, pregnant woman sports a large mandala tattoo above her breast, from which her baby suckles while three children are arranged artfully around her body. A soft backdrop of large trees lends the breastfeeding mother the qualities of Diana, the hunter goddess. The baby and youngest child are both naked while the two older children wear plain cotton underpants and a flimsy shift, adding a Rousseauian state-of-nature touch to the family dynamic.

Nearly all of the outdoor shots construct the mothers as monumental: although contained by, and at one with, their natural surroundings, the mother and baby occupy central stage in a drama that is shot from below, so they appear larger than life. A particularly dramatic shot shows a woman in a red dress, breastfeeding while holding up the traffic, which has come to a standstill behind her while she stands, imperviously facing the camera in the middle of the highway.

Ivens’ indoor shots are opulent, unashamedly celebrating the sensual pleasures of fabric against skin, with gauzy full-length gowns evoking lingerie or evening wear, and lots of flesh on display, while nursing either naked or similarly costumed children and babies. They are posed on expensive lounges belonging in mansions, or reclining on the floor, or — as in one shot — seated in profile on a pedestal in front of an open window. Each occupies their domestic space in an animalistic way highlighting sensuality and luxury. One image is of a woman in a ball gown, but with bare feet; another shows a woman in a sheer cotton shift, lying on the floor in front of the hearth, and stroking a cat with kittens while her breastfeeding child is cradled in her other arm. The association between domesticated mammals playing with their offspring reinforces the strong link Ivens’ work establishes between the natural order of things and breastfeeding.

Conflating the domestic with the natural is not necessarily a retrograde manoeuver, as the women are metonymically connected to their exterior or interior settings as heroines of their domain. Nevertheless, as part of this scenario, it seems important that the images display a breastfeeding woman alone with her child, or if in company, with her other children. This idealization could be interpreted as deeply conservative, implying the maternalist ideal of the breastfeeding woman alone at home. At the same time, the sensuality and power of the figures insist on this being more than an acceptance of duty, by embracing the ‘wild woman’ trajectory of popular feminist works such as Clarissa Pinkola Estes’ *Women Who Run With the Wolves* (1996).

While the images depart from the sanctity and virtue of *Maria Lactans*, they remain anchored to an idealized space, at least at one, if not several, removes from the everyday. The conditions of an intimate public are clear, as the images reveal a sentimental attachment to a mythic fantasy, just beyond reach, but not too far, so they contribute to women’s ‘love affair with conventionality’, operating in ‘aesthetic worlds that are juxtapositional’ to the places where actual change might be realised [48]. Tellingly, the only group image by Ivens is fantastical and painterly, featuring a nativist and countercultural exoticism, replete with elfin children, animals and a shaman figure.

## Tara Ruby

Tara Ruby is a professional photographer and former US veteran who has a reputation for breastfeeding, newborn and maternity portraiture. In recent years she has included women in the military, family photography and performers in concert (who, in contrast to her other work, are all male). While there are some group shots in her online family and military galleries, the breastfeeding shots are purely of mother and child, with the occasional group of women breastfeeding together, as though participating in a well-lit, picturesque nurse-in.

Ruby’s work has been awarded and widely covered in the media, her breastfeeding photos in particular described as ‘stunning’ by *Cosmopolitan* magazine [49]. Partnering with Breastfeeding in Combat Boots, Ruby posted a group shot of 10 active duty women in uniform breastfeeding in 2015, which attracted intense media commentary, and a reprimand from the US Army [50]. In 2016 Ruby followed this up with other portraits of women breastfeeding in uniform, which again attracted controversy and include a woman in firefighting uniform, a Las Vegas showgirl, a nurse and a teacher. Like Ivens’, Ruby is collecting her work into book form.

In contrast to Ivens’ portraits, Ruby focuses on the professional personae of the mothers, combining this element of their identity with their breastfeeding. While some portraits are staged as a personal drama, with soft lighting and pastoral backdrops, the majority show how mothers combine their breastfeeding duties with their professional life, and for this reason are often photographed in uniform or a work environment. Those with a natural backdrop are less staged than Ivens’ images, and more clearly grounded in the everyday, such as that of a woman under a tree on a picnic rug feeding her child, or another woman standing in a field, or sitting in a vineyard, all wearing informal outfits, their babies similarly attired [51]. Here the focus is on the blending of mother and child with her natural surroundings, her enhancement of the space without intrusion. In contrast to the mythic heroinism of Ivens’ portrayals, these women are, sometimes literally, more down to earth.

Ruby’s professional shots are also more striking, with the uniform of the mother, including jacket and helmet, contrasted to the nakedness of the nursing child in the firefighter shot; or in the portrait of a La Vegas show dancer in white sequins and feathered head dress, her child dressed in a white Elvis-style jumpsuit. There is also a school teacher breastfeeding on the floor of the library as she was forbidden from being photographed feeding in the classroom, several nurses and doctors shown feeding in separate portraits, and an airman tandem feeding her sons of different ages, sitting cross-legged on the floor so her combat boots are front and centre.

While these portraits are carefully lit and extremely beautiful, there is less of a fairy tale element here, with more straightforward staging to highlight the juxtaposition of the women’s professional status and their care for their children, making clear the point that the two parts of their lives coexist. As Ruby tells Cosmopolitan.com, “You can work a full-time job and breastfeed and do both equally well…If this is supporting someone out there, then let’s do it” [52].

As with Ruby’s portraits of women in the military, the drama of the other professional images comes from the contrast between the breastfeeding act and the mother’s working identity. While these portraits are either women alone with their child or women nursing in groups, they exhibit a political edge, insisting on the multiple roles of women, and their ability to combine them without detracting from their motherwork.

Ruby’s images open out the breastfeeding imaginary to include other elements of performance beyond mothering. Yet heroinism is also at play here, invoking novelty in the cause of what might be termed ‘extreme breastfeeding’ where athleticism and other forms of daring-do can be associated with breastfeeding as a kind of superpower. Ruby’s work could thus be positioned along a continuum that includes the extraordinary footage of pole dancer Ashley Wright, who posted a video of herself pole dancing and breastfeeding simultaneously, or the image of a naked mother doing a yoga headstand in a garden while her baby lies on the grass suckling her breast. Here there is a release provided by breastfeeding from the everyday, so that it occupies another exotic space beyond the social. Not only are these women alone: they too draw from a mythic frame of reference, except instead of the fairy tales of Ivens’ world, it’s the land of the superhero, where magical thinking makes super-momism possible. As Ashely Wright explains in an interview with *Huffington Post,* “The experience of being able to do an act and live a life that demonstrates strength, balance, sensuality, nurturing, motherhood, power, grace, divine femininity, and then some, all at once, is freeing,” she continued. “It’s my #blackgirlmagic” [53]. Drawing from a similar imaginary of superpowers, Ruby’s breastfeeding mothers in uniform are heroines at rest.

## Nicole McCain and the honest body project

The Honest Body Project is a photographic website set up by the photographer and mother of two, Natalie McCain. Her galleries have expanded over the past few years to include health and wellbeing topics beyond the maternal, such as body image, cancer, loss of a parent, anxiety and depression, in addition to homebirth, c-section, fertility and single mothers. In 2015 McCain photographed a series of mothers with their disabled children and babies. The philosophy behind McCain’s work is to assist women to become more accepting of, and loving towards, their bodies as they experience the changes of maturity, illness, birth and death. Most of the photographs are accompanied by unedited personal stories written by the subjects, ranging from a single, short paragraph to several hundred words. Some of these are interspersed through a series of images creating a narrative that accounts for several years. The section that contains ‘Anonymous accounts of rape culture’ is a more detailed text, with the photographs concealing faces, and some seemingly self-portraits of McCain, in addition to other female figures. The ‘We are 1 in 4: Loss Series’ highlights images with stories by women who have lost a child through miscarriage or infant death [54]. A book of McCain’s work was published in 2017 [55].

The photographs throughout the site are all black and white and the women are usually naked or dressed simply in black underwear, so that the contours of their flesh, and the play of light and shadow across their bodies, is shown in relief against a black background. Similarly, the babies and children are either naked or dressed simply in black shorts, shirts or diapers. Other than the Honest Body Project logo, the background recedes so that the mother’s and child’s bodies float in a kindly lit isolation, lending an air of preternatural innocence. Like Ivens’ and Ruby’s work, the photographs are flattering, designed as momentos, unlike the more confronting visual statements made by the fine art breastfeeding portraiture of Boucher or Opie. By providing a first-person narrative or explanation for the images, McCain’s work is contextualized by the writing rather than by other images or colour. Many of the mothers are shown breastfeeding toddlers as well as children who are old enough to stand to nurse.

Despite the simple staging, the images in the Honest Body Project are arguably more diverse than those of Ruby’s and Ivens’ galleries. This is because the women are shown in a variety of more fluid and active relationships with their children, and often in poses that vary considerably from the more conventional babe-in-arms photos that are featured in most breastfeeding images. As with nurse-in photos, the women in Ruby’s and Ivens’ images tend conform to the standard breastfeeding hold. While this is no doubt the easiest way to take a group shot of women breastfeeding, it reinforces the ideal of the mother holding her baby close to her breast, in an embrace that is controlling and discreet. McCain’s breastfeeding mothers often depart from this pose, sometimes seated while a child stands and feeds, or situated in postures where the child’s agency is made visible.

While there is an element of stylization in the simple lighting and black and white photography, the plain black background in McCain’s work draws the eye to the women’s and children’s naked bodies, and from there to their gestural and physical postures. Without the props of luxury attire, uniforms or picturesque scenery, the women emerge as more playful and agential, as they sit, stand, or lie in a variety of positions, unencumbered by any narrative trappings. If these are ‘action shots’, they reveal a spontaneity that emerges through the act of breastfeeding, demonstrating how it takes on its own narrative through the corporeal communication of skin-to-skin contact and fluid exchange, revealing the body’s surfaces and facial expressions without distraction or obvious framing devices. The mother’s and child’s bodies are revealed to the viewer as a more democratically observed topography — including the child’s face, the mother’s breast and nipple, but equally her arms, legs, face, bottom and torso — which tells a story about the relationship between mother and baby while engaged in feeding. Nursing twins and tandem nursing are also represented. One image shows a woman holding a cloth to her breast and laughing as her baby pulls away. A few drops of milk are visible on his chest. Her text reveals her struggles with oversupply and engorgement.

McCain’s are more expressive images than Ivens’ and Ruby’s, the latter two creating a more glamorous aesthetic, showing women in beautiful clothing or landscapes, and providing a broader framing that draws from nature and landscape photography. McCain’s work is also more overtly political, matched by some of the texts which talk explicitly of the need to normalize breastfeeding in general as well as normalizing breastfeeding in public. The lack of visual framing and absence of colour brings this out, so they work as figurative and documentary statements as much as decorative images.

## Yvette Michelle and Normalize breastfeeding

As stated on its Facebook page, ‘The goal of Normalize Breastfeeding Project is to break stigmas associated with breastfeeding in public and capture local breastfeeding and breastmilk-feeding dyads simultaneously nursing in a single image. These images will be shared in local communities and from the Normalize Breastfeeding Project page’ [56]. Each image is branded with a pink heart-shaped graphic encircling a classic lactation image where the mother-figure’s hair blends into her arms also encircling the baby at the breast. The Normalize Breastfeeding project also organises nurse-ins as photo opportunities such as that organised by the Beaumont Breastfeeding Coalition in 2015, where more than 100 women were photographed from an aerial perspective while grouped into a heart shape. As with other nurse-in or lactivist portraits, the effect of the group shot is double-edged. This is because while it celebrates breastfeeding as a normative activity, and the women are located outside, they are also separated by virtue of being grouped together essentially for this purpose. This reinforces the separateness of breastfeeding mothers from other populations even when outside the home, and for this reason is limited in its usefulness as a means to ‘normalize’ the activity. That the breastfeeding women exist as a group also lends an unfortunate ‘cargo cult’ element to their beliefs and practice: the implication is that they are banded together against those who disapprove. While this is in a sense true, given the rhetoric of public protest, it detracts from the need for mothers to become integrated *within* their publics, to be truly at ease while breastfeeding. It also sends a mixed message, for while their performance of breastfeeding is deeply conservative in terms of embodying the classic breastfeeding hold in discreet poses, the performativity intrinsic to the shot reinforces the suspicion that the women are exhibiting themselves to provoke, rather than to feed their offspring. This raises the question of whether this is strategically effective, when those who disapprove of breastfeeding already feel provoked by a mother alone with her baby in a café or shopping centre, discreetly, silently, unobtrusively nursing?

It is a perhaps a unique difficulty for breastfeeding advocacy, since the activity comes up against conservative views of women’s proper habitation of public spaces [20]. While they remain proper while in the ‘group’, does that propriety extend to them breastfeeding alone in public? It’s hard to imagine that those who are unsympathetic in the first place would change their mind on seeing these images. The term ‘exposure therapy’ is adopted by the Beaumont organiser, and it could also be said that these images fulfil a consciousness-raising or therapeutic function for other breastfeeding women. But the logistics of having women breastfeed together in groups means that the staging of these events detracts from their ‘naturalness’ or incidental inevitability, which is precisely the image needed for women to feel safe while breastfeeding alone, in public [57].

Its redeeming feature may be that the event provides a social occasion for the women involved.

Another kind of group shot appears in a World Breastfeeding Photo, also from 2015, where two photos feature a pair of women of Caucasion and African American appearance sitting together on steps in a garden, feeding their children, including one who is tandem feeding. Yet while they are socialising with each other, they are still separated from others. The opposite of the performative outdoor group shots, these images imply the need to hide while feeding outside. The secretive nature of their partnership, and the absence of others in the frame, underscores the necessity for discretion.

## Conclusion

Breastfeeding portraiture of the kinds discussed here, in their different ways invoke an audience for breastfeeding and bring it into being as a public event worthy of aesthetically framed appreciation. Rather than merely an act inviting surveillance and moral judgement, they provide a form of showcasing that asserts the legitimacy of the act and its potential for being staged to appeal to the viewer beyond its utilitarian or dutiful meanings. Attention to the aesthetic potentials of breastfeeding, also enables the viewer to consider breastfeeding as a form of pleasure [58] as well as considering its many possible meanings ‘in relation to itself’ rather than ‘by reference to a criterion of utility’ [59]. By ramping up the glamour of breastfeeding, Ivens work in particular also offers a radical reminder of breastfeeding’s autoerotic sensuality. Likewise, Ruby’s women in uniform epitomize the self-sufficiency of breastfeeding women, their professional personae underscoring a maternal competence that is mutually reinforcing of their identity as working mothers. Finally, McCain and Michelle’s work represents a more accessible and realizable scenario for breastfeeding where an audience acknowledges that breastfeeding involves the exposure of the body (McCain) and may occur in a range of everyday suburban settings (Michelle).

A common element of these portraits by Ivens, Ruby, McCain and Michelle is that the mothers and babies are most often portrayed at a proxemic distance from the viewer. As an aesthetic strategy, this opens up a variety of visual possibilities from a photographer’s perspective, enabling such elements as the low horizon which emphasizes a monumentalism in the mother’s stature, or a depth of field that emphasizes the mother’s and child’s continuity with a dramatic or verdant landscape. Yet again, this also situates the breastfeeding mother at a literal distance signifying inaccessibility. The act of breastfeeding may attain painterly layers of meaning in these portraits, which may intrigue or intimidate, as much as welcome the viewing public. While indubitably gorgeous, the implications of this distancing effect for the practice of breastfeeding are multiple: it’s harder to achieve; it’s more exceptional than quotidian; it’s more mythical than commonplace; its continuity with a natural landscape may divorce it from the social; and it’s rarefied, which, like the natural background itself, is in many ways understood to be at risk of extinction.

The diversity and volume of images of women breastfeeding in public (as well as in domestic spaces) has increased exponentially over the past decade. The role of digital media in the wide circulation of these images, at least among other women, has provided a unique opportunity for the participatory condition of these women to be enhanced beyond solitude, even though they may be alone while doing so. Added to this, the interest in images of breastfeeding, fuelled by celebrity brelfies in mainstream women’s magazines, and the rise of breastfeeding-positive professional photographers, has provided the opportunity for advocates, mommy bloggers and other enthusiasts to collect artwork and more obscure social history images of women breastfeeding in a variety of circumstances. At the same time, the opportunity to represent mothers breastfeeding while also socializing with other adults and children, in quotidian circumstances, spaces and conditions, remains elusive. Until this changes, breastfeeding will continue to belong in a space of seclusion, either idealized within the fantastical realm of the mythic maternal, or politicized as lactivist separatism in group protest shots, and in both instances held at a reverential distance from everyday social interactions with others.

By creating an intimate public, the existing range of breastfeeding portraiture both supports and detracts from efforts to normalize breastfeeding. Ivens’ and Ruby’s work differentiates the breastfeeding mother from the everyday, as a mythic, larger than life maternal heroine, or as a professional supermom/superhero. McCain’s work offers a more intimate understanding of the embodied experience of breastfeeding, but despite their loving detail, the figures are isolated against the black backdrop, which acts as a kind of Platonic neverland, beyond place or space. Familiarity with the health benefits of breastfeeding over formula, as well as social support and ‘comfort with breastfeeding in social settings’ is a predictor of exclusive breastfeeding, yet opportunities to achieve this familiarity through the open circulation of diverse images remain limited [60–62].

While brelfies in particular have encouraged women to share images of themselves breastfeeding from their own perspective, and as a performative activity for communication, the opening out of breastfeeding practice into spaces of actual social interaction is yet to be represented. Like brelfies, the breastfeeding portraiture examined in this article highlights the singularity of the breastfeeding dyad: the sociality of the mothers and their children in these images remains at a proxemic distance from their audience. The conjuring of an audience of any kind is an advance on complete concealment, secrecy and shame. Yet in permitting visualization within strictly decorous and conventional poses, or for strictly advocacy purposes, most images of breastfeeding mothers and children remain isolated and distanced from the everyday. As such, the breastfeeding mother remains an exotic figure, excluded from participating in most social interactions and occasions. As long as the otherness of breastfeeding remains intact, she will remain at bay, in both discursive and lived spaces of contestation.

## Footnotes

1 It should be noted that a breastfeeding mother is never actually solitary since she is by definition in the company of her baby or babies, and for this reason is always already social. The definition of sociality here instead signifies adult company implying a sense of audience as well as companionship. See my article ‘Making breastfeeding social’ for further discussion of this point [27].

2 Rare exceptions exist in photographs produced by the natural home birth movement in the 1970s but these have not been digitized and are not readily available outside deposit libraries.

3 I haven’t seen any examples of group breastfeeding selfies, and the mechanics of crowding into the frame while breastfeeding could make this difficult.
